# Selective effects of a therapeutic protein targeting tumor necrosis factor-alpha on cytochrome P450 regulation during infectious colitis: implications for disease-dependent drug–drug interactions

**DOI:** 10.1002/prp2.27

**Published:** 2014-02-12

**Authors:** Beatrice A Nyagode, Roya Jahangardi, Matthew D Merrell, Malú G Tansey, Edward T Morgan

**Affiliations:** 1 Department of Pharmacology, Emory University School of Medicine1510 Clifton Road NE, Atlanta, Georgia, 30322; 2 Department of Physiology, Emory University School of Medicine1510 Clifton Road NE, Atlanta, Georgia, 30322

**Keywords:** Biologics, cytochrome P450, drug metabolism, drug–drug interactions, inflammation, therapeutic proteins

## Abstract

We studied the impact of administering XPro1595, a novel antagonist of soluble tumor necrosis factor-*α*(TNF*α*), on the regulation of hepatic cytochrome P450 enzymes in the *Citrobacter rodentium* model of infectious colitis. XPro1595 was administered subcutaneously every 3 days throughout the infection, or as a single injection near the peak of infection. When given throughout the infection, XPro1595 selectively blocked the downregulation of Cyp3a11 and 3a25 mRNAs, as well as the induction of Cyp2a4/5, without affecting the downregulation of Cyp4a10, Cyp4a14, Cyp2b10, or flavin-mooxygenase-3. Induction of Cyp3a11, Cyp3a25, Cyp2c29, and Cyp3a13 mRNAs were observed only in XPro1595-treated mice. Administration of a single dose of XPro1595 was relatively ineffective. These results (1) confirm the role of soluble TNF*α* in hepatic Cyp3a regulation during infectious colitis deduced from studies in TNF*α* receptor-1 knockout mice; (2) indicate the potential for soluble TNF*α* -specific antagonists to cause disease-dependent drug–drug interactions; and (3) suggest a novel mechanism by which an anti-inflammatory therapeutic protein can produce an opposite effect to that of the disease by selectively neutralizing one of multiple signals regulating drug-metabolizing enzyme expression. More research is needed to determine whether or not this is applicable to other diseases or disease models.

## Introduction

Infections and inflammation cause changes in the activity and expression of many drug-metabolizing enzymes including members of the cytochrome P450 (P450) superfamily (Aitken et al. 2006). In most cases, this results in reduced drug clearance and increased exposure to drugs that are substrates for these enzymes. Cytokines such as interleukin (IL)-6, interferon (IFN)*α* or *γ*, and tumor necrosis factor (TNF)*α* are thought to mediate the effects of inflammation on P450 regulation. While it has been shown that such cytokines can regulate P450 expression in hepatocyte cultures (Abdel-Razzak et al. 1993; Chen et al. 1995; Tapner et al. 1996; Aitken and Morgan 2007; Dickmann et al. 2012) and in vivo (Renton et al. 1984; Ghezzi et al. 1986a,b; Morgan et al. 1994), the roles of individual cytokines on regulation of drug metabolism in different diseases are not well understood.

The need to understand which cytokines are involved in P450 regulation in vivo is sharpened by the recently discovered phenomenon of disease-dependent drug–drug interactions (DDDI), in which therapeutic proteins (biologic drugs) targeted toward cytokines or their receptors can affect the metabolism of small molecule drugs by reversing the downregulation of P450 enzymes caused by the inflammatory disease, as reviewed in (Morgan 2009). This was first demonstrated by the attenuation of Cyp3a downregulation in mice by a polyclonal antibody to IL-6 in a genetic model of arthritis (Ashino et al. 2007). Cyp3a downregulation in a different, preadjuvant model of arthritis was inhibited by the anti-TNF*α* biologic infliximab (Ling and Jamali 2009). Subsequently, the anti-IL-6 receptor antibody tocilizumab was shown to increase clearance of the CYP3A substrate simvastatin in humans with rheumatoid arthritis (Schmitt et al. 2011). A recent white paper on the subject illustrates both the clinical and regulatory concerns for DDDIs and the need for more information on cytokine regulation of P450s during inflammatory disease (Evers et al. 2013).

In addition to the study using infliximab described above, four other studies have directly tested the in vivo role of TNF*α* in P450 regulation in a disease model. The downregulations of Cyp1a, 2b, 3a, and 4a following bacterial lipopolysaccharide (LPS) injection were not attenuated in mice deficient in both TNF*α* receptor-1 (TNFR1) and TNFR2, (Warren et al. 1999), whereas the responses of Cyp2d and Cyp2e1 enzymes were attenuated. In agreement with this finding, Cyp3a11 and 2c29 downregulations by LPS were unaffected in TNF*α*-null mice (Ashino et al. 2004) (Miyoshi et al. 2005). In contrast, downregulation of Cyp3a11 and Cyp2c29 by injection of tuberculosis vaccine was somewhat attenuated in TNF*α*-deficient animals (Ashino et al. 2004). Previous studies by our laboratory (Kinloch et al. 2011) have demonstrated that TNF*α* is an important factor in selectively regulating the expression of P450s of the Cyp3a subfamily in *Citrobacter rodentium*-induced colitis. *C. rodentium* is a noninvasive rodent pathogen equivalent to human enteropathogenic *Escherichia coli*, a serotype of *E. coli* that causes colitis in humans (Higgins et al. 1999). The colitis caused by the bacteria is characteristic of inflammatory bowel disease (Higgins et al. 1999). Cyp3a11 and Cyp3a25 were significantly downregulated in *C. rodentium*-infected C57BL/6 mice, while the downregulation was inhibited in TNFR1 knockout mice (Kinloch et al. 2011). Hepatocyte experiments found that TNF*α* was the most potent and efficacious cytokine tested in the downregulation of Cyp3a enzymes and other P450s in mouse hepatocyte cultures (Nyagode et al. 2010; Kinloch et al. 2011). Together, these results suggest a role for TNF*α* in the regulation of Cyp3a enzymes in vivo, which is dependent on the specific disease or disease model.

However, the lack of Cyp3a downregulation observed in TNF*α*- or TNFR-null mice could be due to adaptive changes in the mouse due to the long-term absence of the cytokine or its receptors rather than to blockade of TNF*α* effects. This possibility can be addressed using a pharmacological approach to block TNF*α* action in wild-type mice. The biologic drugs currently in clinical use do not discriminate between soluble or membrane-bound forms of TNF*α*, which signal through different receptors (Holtmann and Neurath 2004). We therefore conducted a study using a biologic drug that selectively neutralizes soluble TNF*α* to eliminate the potential influence of these adaptive changes. We used XPro1595, a dominant-negative form of TNF*α* (Y87H, A145R) that forms heterotrimers with native soluble TNF*α* to give complexes that neither bind to nor stimulate signaling through TNF*α* receptors (Steed et al. 2003; Zalevsky et al. 2007). This experiment was designed to test XPro1595's ability to block or reverse the effects of *C. rodentium* infection on hepatic cytochrome P450 enzymes, with a focus on the Cyp3a subfamily. The results confirmed the role of TNF*α* in Cyp3a downregulation in *C. rodentium infection*, and demonstrated the potential of a dominant-negative biologic to produce disease-dependent interactions with small molecule drugs via Cyp3a regulation.

## Materials and Methods

### Bacteria

*Citrobacter rodentium* wild-type strain (51116) was received from the American Type Culture Collection (Manassas, VA), and grown overnight in Luria broth at 37°C without shaking. Bacterial growth was monitored by spectrometry at 600 nm and the optical density was used to calculate a nominal bacterial dose of 2.5 × 10^8^ cfu (colony forming units) per mouse, assuming intake of 8 mL/day per mouse. Actual concentrations of bacteria were determined by retrospective plating on MacConkey agar. After bacterial administration, the remaining drinking water volume was recorded to allow calculation of the actual bacterial dose.

### Chemicals, animals, and treatments

All reagents and chemicals were obtained from Sigma-Aldrich (St Louis, MO) unless otherwise specified. Thirty-six female C57BL/6J mice were obtained from Jackson Laboratory (Bar Harbor, ME), of which 18 were controls and 18 were experimentally infected. The mice were housed in six separate groups of six to a cage and acclimatized to the animal facility for 1 week before the beginning of the experiment. The mice were 9 weeks of age when injections started.

**Figure 1:**
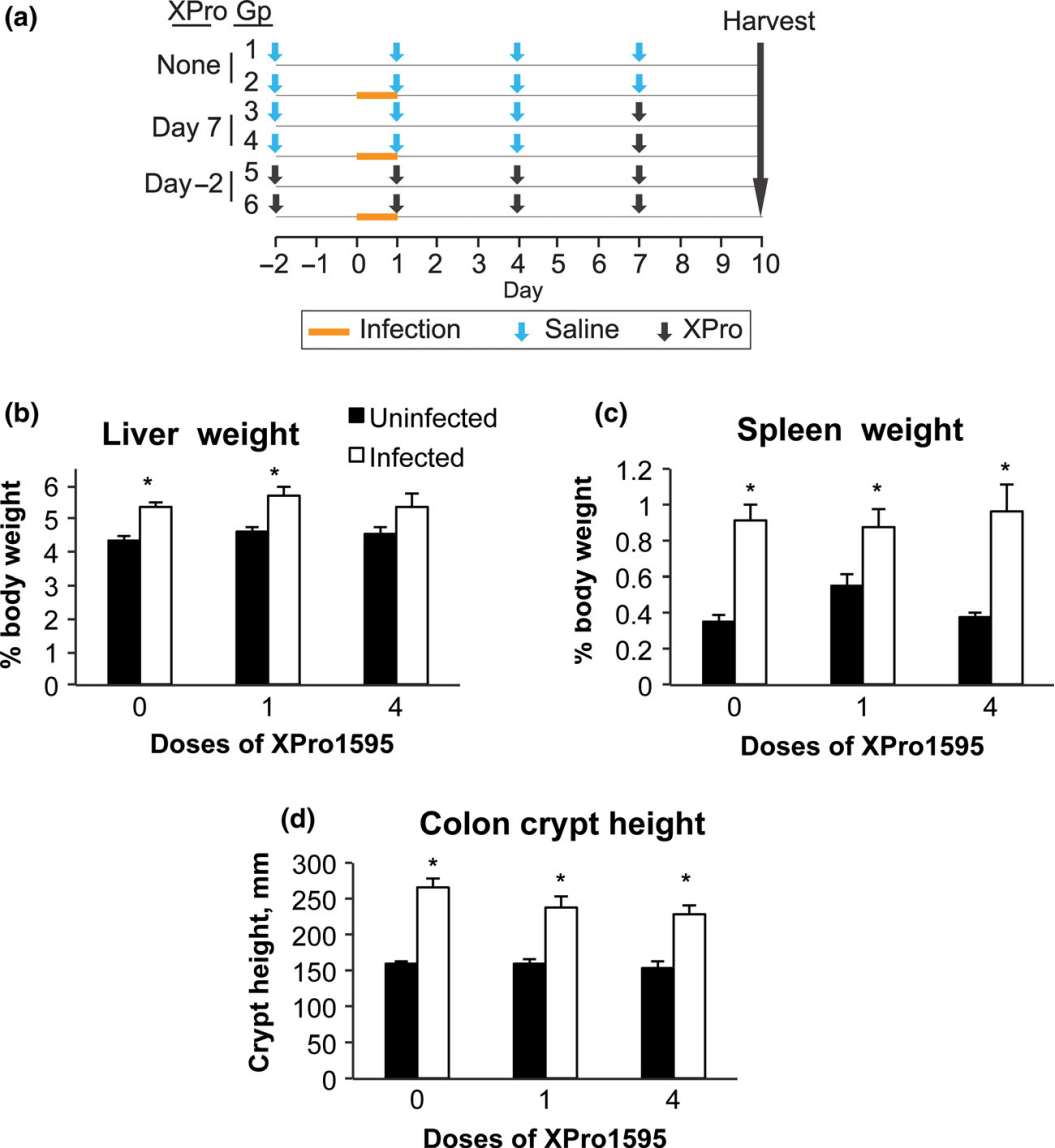
Experimental design, organ weights, and colon crypt heights. Animals were treated as described in Materials and Methods, and euthanized 10 days after infection. (A) Schematic representation of the experimental design. (B) Liver weights. (C) Spleen weights. (D) Colon crypt heights. Values are the mean tissue weight after normalization to body weight ± SEM (*n* = 6). *Significantly different from uninfected animals in same XPro1595 treatment group, *P* < 0.05, Student's *t*-test (or *U*-test if variances were not equivalent).

A diagrammatic view of the treatment protocol is presented in Figure 1A. Infection started on day 0 when groups 2, 4, and 6 were orally administered *C. rodentium* in their drinking water (containing 20% sucrose) for 24 h at a nominal dose of 2.5 × 10^8^ cfu per mouse drinking 8 mL/day. Control animals received 20% sucrose in water without bacteria during the same time period. Infected mice were housed in a biosafety level 2 facility to prevent transmission. Food was withheld during the 24-h infection period. TNF*α* antagonist XPro1595 (Xencor Inc., Monrovia, CA) was injected subcutaneously at a dose of 10 mg/kg in saline. Groups 1 and 2 (No XPro1595) received saline injections on days −2, 1, 4, and 7. Groups 3 and 4 (1 XPro1595 dose) were injected with saline on days −2, 1, and 4, and received a single injection of XPro1595 on day 7, 3 days prior to sacrifice. Groups 5 and 6 (4 XPro1595 doses) received injections on days −2, 1, 4, and 7. Animals were sacrificed by decapitation under isoflurane anesthesia on day 10. The Institutional Animal Care and Use Committee of Emory University approved all animal procedures.

### Tissue collection

Livers were collected and rinsed in cold 1.15% potassium chloride. They were then weighed, portioned, flash-frozen in liquid nitrogen, and stored at −80°C for later RNA preparation. Blood was collected after decapitation and allowed to clot. Serum was separated by centrifugation for 10 min at 7500*g* and stored at −80°C for later analysis. Spleen was collected and rinsed in 1.15% potassium chloride and weight was recorded. Colon was removed and washed of fecal matter with cold 1.15% potassium chloride. The distal quarter of the colon was dissected and kept on ice for later detection of viable bacteria. The next most distal portion was used for histological analysis.

### Determination of tissue bacterial loads

Liver and colon samples were weighed and homogenized (Ultra Turrax T25, IKA Wilmington, NC) in 1 mL of phosphate-buffered saline. The colon homogenates were diluted 10^4^- or 10^5^-fold and 50 *μ*L was plated onto separate MacConkey agar plates. Liver homogenates (100 *μ*L) and blood (50 *μ*L) were plated directly. The plates were incubated for 16 h at 37°C and colonies were counted.

### Colon crypt length measurement

Distal colon sections were fixed in 10% formalin and embedded in paraffin. Five *μ*m sections were cut and stained with hematoxylin and eosin. The heights of three well-oriented crypts were measured in the distal colon for each mouse, using a Zeiss 200M microscope (Thornwood, NY) with a 20× NA1.4 lens, and Slidebook (Intelligent Imaging Innovations, Denver, CO). The measurements were made in a blinded fashion.

### RNA extraction, cDNA synthesis, and real-time polymerase chain reaction

Total liver RNA was prepared using RNA-Bee isolation reagent (Tel-Test Inc., Friendswood, TX), as directed by the manufacturer. The extracted RNA was analyzed on an agarose gel in order to determine RNA purity and integrity following visualization with ethidium bromide. RNA concentration was determined spectrophotometrically by measuring absorbance at 260 nm.

Relative expression of liver mRNAs was measured using reverse transcriptase – real-time polymerase chain reaction (RT-qPCR). Purified total RNA was reverse-transcribed with a SuperScript First-Strand Synthesis System Kit (Invitrogen/Life technologies, Carlsbad, CA) according to the manufacturer's protocol. Primers (Table S1) were custom-synthesized by MWG Biotech, Inc. (High Point, NC) or Operon Biotechnologies Inc. (Huntsville, AL). Real-time PCR was performed using Power SYBR Green Master Mix reagent (Applied Biosystems/Life Technologies, Carlsbad, CA) and an Applied Biosystems PRISM 7000 sequence detection system. Duplicate reactions were performed using a total volume of 25 *μ*L with Power SYBR Green Master Mix reagent (Applied Biosystems) along with forward and reverse primers of choice. The thermal cycling program included 2 min at 50°C, 10 min at 95°C, followed by 40 cycles of 95°C for 15 sec, and 1 min at the primer's own annealing temperature. Results were expressed as relative levels of target mRNA. The housekeeping gene glyceraldehyde-3-phosphate dehydrogenase (GAPDH) was used to normalize the expressed mRNA levels by the ΔΔCt method (Livak and Schmittgen 2001), after verifying that GAPDH levels were not affected under the experimental conditions. The expression levels in the uninfected, untreated controls were arbitrarily set at 1.

### Microsomal preparation and western blotting

All procedures were done at 4°C. Livers were homogenized in four volumes of Tris acetate buffer, pH 7.4 containing 1 mM ethylenediaminetetraacetic acid (EDTA) and 0.1 mol/L KCl. Homogenates were centrifuged at 7500*g* for 25 min, and the supernatant was then centrifuged for 35 min at 250,000*g*. The microsomal pellet was resuspended in 10 mmol/L Tris acetate buffer, pH 7.4, containing 0.1 mmol/L EDTA and 23% glycerol, and the final microsomal solution was stored at −80°C. Protein concentrations were determined with a bicinchoninic acid protein assay kit (Thermo Fisher Scientific, Waltham, MA) with bovine serum albumin as the protein standard.

Western blotting was used to measure relative levels of P450 protein levels in mouse hepatic microsomes as described previously (Chaluvadi et al. 2009; Kinloch et al. 2011). Antibodies to rat CYP3A and to NADPH-cytochrome P450 reductase (Cpr) were generously provided by Dr. James Halpert (University of California, San Diego) and Dr. Bettie Sue Masters (University of Texas, San Antonio). Antibody to CYP4A (ab140635) was purchased from Abcam (Cambridge, MA). All antibodies above were diluted 1:5000 for detection, and the assays were performed within a linear range of protein loaded. Proteins were detected using a SuperSignal West Pico chemiluminescent substrate kit (Thermo Fisher Scientific) where the bands were visualized by fluorography on x-ray film and then digitized. The bands were quantified by densitometry using Kodak Molecular Imaging software (Eastman Kodak Co., Rochester, NY).

### Cytokine analysis

Serum samples were subjected to multiplexed immunoassays by the Emory Multiplexed Immunoassay Core (EMIC), using a mouse proinflammatory 7-PLEX kit (Meso-Scale, Rockville, MD) and the Discovery Sector 2400 instrument (Meso-Scale). Samples were analyzed for cytokines IFN*γ*, TNF*α*, chemokine (C-X-C motif) ligand 1 (CXCL1, mKC), IL1, IL6, IL10, and IL12.

### Statistical analysis

One-way analysis of variance (ANOVA) was used to determine if significant differences existed among infected groups in tissue bacterial colonization. For all other parameters, two-tailed independent Student's *t*-test was used to determine the difference between uninfected and infected groups, where the level of significance was set at *P* < 0.05. Log transformation was performed in cases where the variances were not equivalent. If the variances of the transformed data were still not equivalent, the Mann–Whitney *U*-test was used.

## Results

### Experimental design

This experiment was designed to test whether a single XPro1595 injection administered when downregulation is already established could reverse (Single-injection groups), and whether TNF*α* inhibition by XPro1595 throughout the experiment (four injections groups) could block, the downregulation of CYP3a family members. A summary of the experimental protocol is shown in Figure 1A. We determined previously that the downregulation of P450 enzymes is optimal between 7 and 10 days after infection (Chaluvadi et al. 2009). The dose and dose interval for XPro1595 were chosen because they were demonstrated to promote functional recovery in a model of experimental autoimmune encephalitis (Brambilla et al. 2011).

### Tissue weights, colon crypt heights, and tissue bacteria in *C. rodentium*-infected mice

Throughout the experiment, none of the mice showed obvious clinical signs of illness. However, infected mice had slightly looser feces compared to control mice. All animals survived the duration of the *C. rodentium* infection and no significant changes in body weight were found (data not shown). There were no significant differences in bacterial counts in the colon, liver, or blood (Fig. S1). Infected mice had a 21% increase in liver weight and this was slightly reduced in the animals receiving XPro1595 throughout the experiment (four injections, Fig. 1B). As expected, spleen weights were dramatically increased by infection (2.5-fold) and this was the same with or without XPro1595 treatment (Fig. 1C). Colon crypt heights, a measure of mucosal hyperplasia, were significantly increased by 67%, 47%, and 47%, respectively, in the 0, 1, and 4 dose groups (Fig. 1D).

### Effects of TNF-*α* antagonist on regulation of hepatic P450 mRNAs and proteins during infection

**Figure 2:**
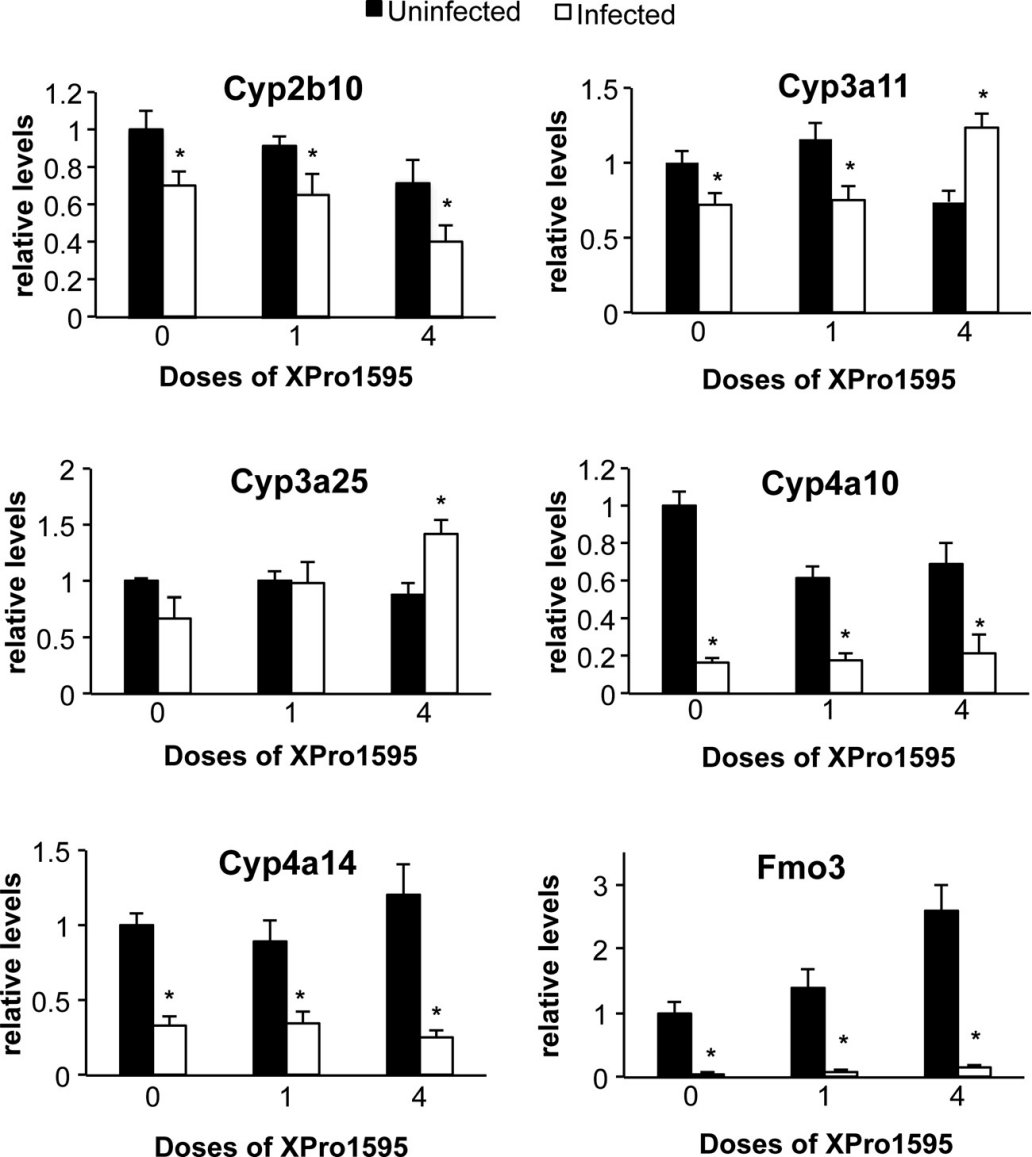
Effect of TNF-*α* antagonist on downregulated P450 and Fmo3 mRNAs following *Citrobacter rodentium* infection. Relative levels of mRNA were measured by RT-qPCR with normalization to GAPDH mRNA, and are expressed relative to the levels in uninfected mice without XPro1595 treatment, which was arbitrarily set at 1. Values represent mean ± SEM. (*n* = 6). *Significantly different from uninfected animals in same XPro1595 treatment group, *P* < 0.05.

We chose to study a panel of P450 enzymes that we have characterized extensively in this infection model (Chaluvadi et al. 2009; Nyagode et al. (2010); Kinloch et al. 2011). They represent the major subfamilies of drug-metabolizing P450s, as well as Cyp4 family enzymes that have roles in metabolism of fatty acids and eicosanoids. *C. rodentium* infection alone caused significant downregulation in the following hepatic P450 mRNAs: Cyp4a10 (to 14% of control), Cyp4a14 (34%), Cyp3a11 (72%), Cyp2b10 (70%), and Fmo3 (6%) (Fig. 2). While decreases in the mean values for mRNA expression of Cyp3a25 (67%, *P* = 0.10, Fig. 2) and Cyp2d22 (not shown) were observed, they did not achieve statistical significance. Treatment with four doses of XPro1595 beginning on day −2 (groups 5 and 6), but not with a single dose on day 7 (groups 3 and 4), not only blocked Cyp3a11 downregulation, but actually reversed it so that an increase was observed (Fig. 2). The same pattern was seen with Cyp3a25. In contrast, the downregulation of other P450s and Fmo3 were unaffected by any XPro1595 treatment (Fig. 2).

**Figure 3:**
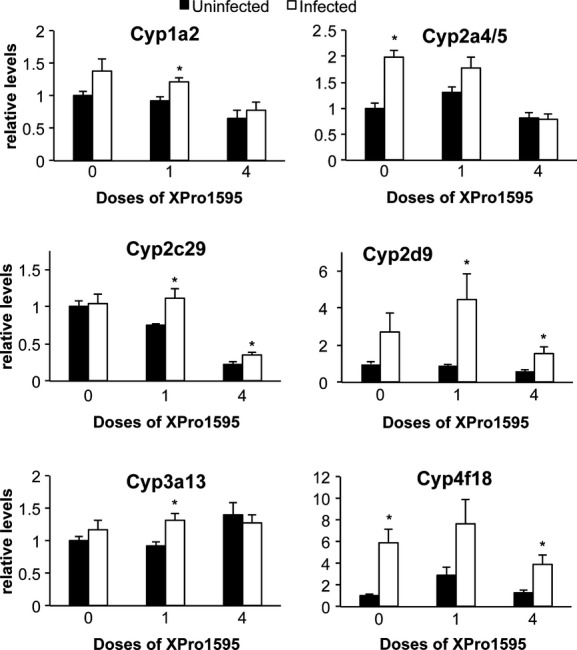
Effect of TNF-*α* antagonist on upregulated P450 mRNAs following *Citrobacter rodentium* infection. Relative levels of mRNA were measured by RT-qPCR with normalization to GAPDH mRNA, and are expressed relative to the levels in uninfected mice without XPro1595 treatment, which was arbitrarily set at 1. Values represent mean ± SEM. (*n* = 6). *Significantly different from uninfected animals in same XPro1595 treatment group *P* < 0.05.

**Figure 4:**
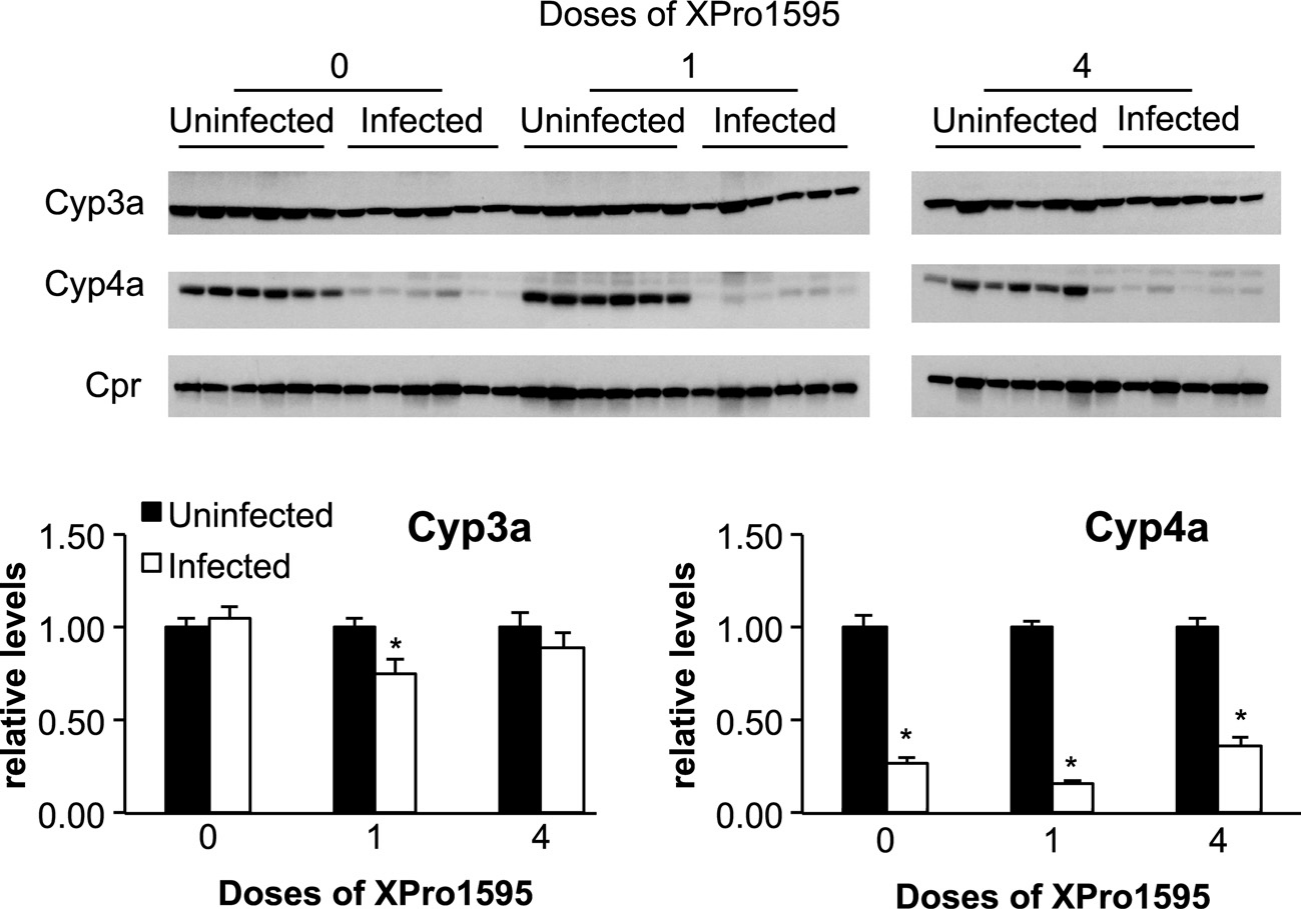
Effect of TNF-*α* antagonist on P450 protein expression following *Citrobacter rodentium* infection. *Upper panels*: Representative Western blots for Cyp3a, Cyp4a, and Cpr. Equal amounts of protein were loaded in each lane. *Lower panels:* Quantitative analyses of the data. Cyp3a and Cyp4a protein band intensities were normalized to the intensity of Cpr, used here as a loading control. Protein levels are expressed relative to the levels in uninfected mice without XPro1595 treatment, which was arbitrarily set at 1. Values represent mean ± SEM. *Significantly different from uninfected animals in same XPro1595 treatment group, *P* < 0.05, *n* = 6.

*Citrobacter rodentium* infection tended to induce several other P450 mRNAs (Fig. 3). Cyp2a4/5 (2.0-fold), Cyp4a12 (8.2-fold), and Cyp4f18 (5.9-fold) were significantly induced by infection (Fig. 4). Cyp2d9 (Fig. 3, *P* = 0.06) and Cyp1a2 (*P* = 0.11, not shown) showed trends toward induction that were not statistically significant. The increase in Cyp2a4/5 was attenuated when XPro1595 was injected during ongoing infection (one dose), and blocked when XPro1595 was administered throughout the infection (four doses) (Fig. 3). Cyp2d9, although significantly induced by infection in the single-dose group, was less induced in the group receiving four doses, suggesting a dose-dependent effect of XPro1595 (Fig. 3). The induction of Cyp4f18 (Fig. 3) and Cyp4a12 (not shown) was minimally affected by XPro1595. Cyp1a2, Cyp3a13, and Cyp2c29 were each induced significantly in the group that received one dose of XPro1595 group, whereas only trends toward induction were observed in the group without XPro1595 (Fig. 3).

Western blotting was used to determine whether the changes in mRNA expression also manifested in changes in protein levels. Cpr protein levels were not affected by infection, and were used as a loading control. As seen in Figure 4, the effect of XPro1595 treatment on Cyp4a proteins was consistent with the effects observed on Cyp4a10 and 4a14 mRNAs (Fig. 2). That is, Cyp4a proteins were downregulated to 14% of control during infection, and this was unaffected by XPro1595 treatment. However, infection failed to affect steady-state levels of Cyp3a proteins detected by this antibody.

### Effects of TNF-α antagonism on hepatic cytokine and acute phase protein (APP) mRNA expression, and serum cytokine levels during C. rodentium infection

**Figure 5:**
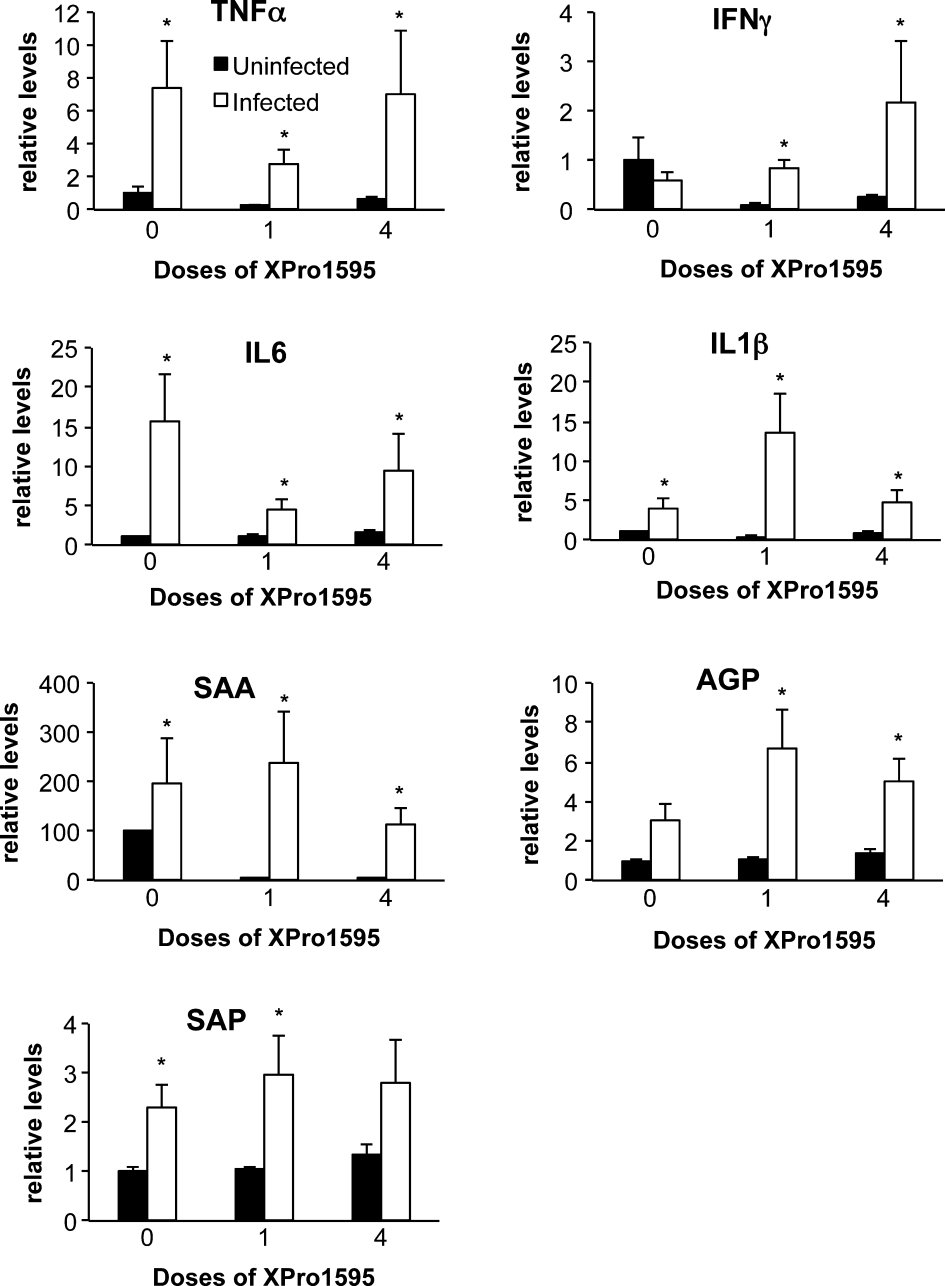
Hepatic cytokine and acute phase protein mRNAs in mice treated with XPro1595. Relative levels of mRNA were measured by RT-qPCR with normalization to GAPDH mRNA, and are expressed relative to the levels in uninfected mice without XPro1595 treatment, which was arbitrarily set at 1. Values represent mean ± SEM. (*n* = 6). *Significantly different from uninfected animals in same XPro1595 treatment group, *P* < 0.05.

Infection with *C. rodentium* produced significant increases in the hepatic mRNA expression of TNF*α*, IL6, IL1*β*, serum amyloid A (SAA), and serum amyloid P (SAP), indicative of a hepatic inflammatory response (Fig. 5). IFN*γ* and *α*_1_-acid glycoprotein (AGP) were not significantly affected, although AGP showed a trend toward induction. A single XPro1595 treatment on day 7 attenuated the induction of TNF*α* and IL6, but had little effect on the induction of the acute phase proteins SAA, AGP, and SAP. Interestingly, with the exception of IFN*γ*, these effects of XPro1595 were smaller or absent when four doses were administered beginning on day −2 (Fig. 5).

**Figure 6:**
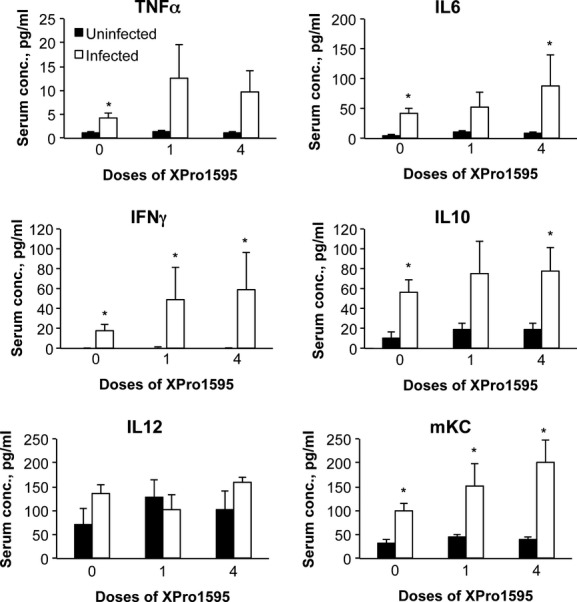
Effect of the TNF-*α* antagonist XPro1595 on serum cytokine levels. Blood was harvested at sacrifice and serum was prepared and analyzed by multiplexed immunoassays. Values represent mean ± SEM. (*n* = 6). *Significantly different from uninfected animals in same XPro1595 treatment group, *P* < 0.05.

Serum cytokines (Fig. 6) showed a different pattern of regulation than hepatic cytokine mRNAs, indicating that the liver is not the major source of circulating cytokines during *C. rodentium* infection. XPro1595 treatment did not attenuate any of the observed increases in serum cytokines (Fig. 6). The increases in circulating IL10 and TNF*α* did not appear to be affected by XPro1595 (Fig. 6). Serum IL1 was unaffected by infection in any of the groups (not shown).

## Discussion

In this study, the soluble TNF*α* antagonist XPro1595 selectively antagonized the downregulation of Cyp3a11 and 3a25, as well as the upregulation of Cyp2a4/5 during C. *rodentium* infection, an acute inflammatory disease. Other downregulated transcripts including Cyp2b10, Cyp4a10, Cyp4a14, and Fmo3 were unaffected by XPro1595 treatment. These data implicate an in vivo role for soluble TNF*α* in downregulating certain Cyp3a genes in this inflammatory disease model. It also suggests that XPro1595 is in principle capable of causing DDDI via Cyp3a modulation in this particular model. Ling and Jamali (2009) first demonstrated the potential for TNF*α* DDDI using infliximab in a rat preadjuvant arthritis model. However, no such DDDI has yet been reported for TNF*α* in humans. It remains to be determined whether or not TNF*α* antagonism will reverse Cyp3a downregulation in models of chronic inflammatory disease, for which biologic drugs are prescribed in humans.

The selective blockade of Cyp3a downregulation by XPro1595 is in excellent agreement with the findings in TNFR1 knockout mice and in cultured hepatocytes (Kinloch et al. 2011). These mice show increased pathologic responses to *C. rodentium infection* (Goncalves et al. 2001), and accordingly they had increased hepatic expression of TNF*α*, IL6, IL1*β*, and AGP indicative of an increased inflammatory response (Kinloch et al. 2011). As a result, they actually had potentiated downregulation of Cyp4a10, Cyp4a14, and Fmo3, as well as potentiated induction of Cyp2d9 and Cyp4f18 (Kinloch et al. 2011). XPro1595 did not attenuate the hepatic or serum cytokine responses to infection (Figs. 5 and 6) and there was no significant impact of XPro1595 on spleen weight or on tissue levels of bacteria. Thus, a generalized decrease in inflammation is not responsible for XPro1595's reversal of Cyp3a downregulation nor its blockade of Cyp2a4/5 induction. Because XPro1595 neutralizes only the soluble, circulating form of TNF*α* (Zalevsky et al. 2007) which signals primarily through TNFR1 (Holtmann and Neurath 2004) we can conclude that soluble TNF*α* regulates Cyp3a expression in this disease model, and not the membrane-bound form of TNF*α,* which signals primarily through TNFR2 (Grell et al. 1995). Future studies should compare and contrast the effects of XPro1595 in this model with those of biologics that target TNF*α* nonselectively (e.g., etanercept).

In this study, infection by *C. rodentium* failed to significantly affect Cyp3a protein expression as measured by Western blotting. We have previously observed that changes in Cyp3a proteins during infection are smaller in magnitude than changes in Cyp3a mRNAs (Nyagode et al. 2010). This could be explained by kinetic considerations, posttranscriptional regulation, by the unknown specificity of the antibodies (raised against rat CYP3A) for mouse enzymes, or by a combination of these factors.

Our qPCR primers could not distinguish Cyp2a4 and 2a5 mRNAs, which have 96% sequence identity (Lindberg et al. 1989). It seems most likely that our observations represent an increase in Cyp2a5 mRNA, which is known to be induced under several pathophysiological conditions (Su and Ding 2004). Cyp2a4/5 mRNA expression was increased by infection, and this effect was abolished by XPro1595 given from day −2 indicating a role for TNF*α* in the induction of Cyp2a4/5 during *C. rodentium* infection. There was a tendency toward the same phenomenon for Cyp1a2, but this could not be determined because the “effect” in the XPro1595 controls was not significant.

**Figure 7:**
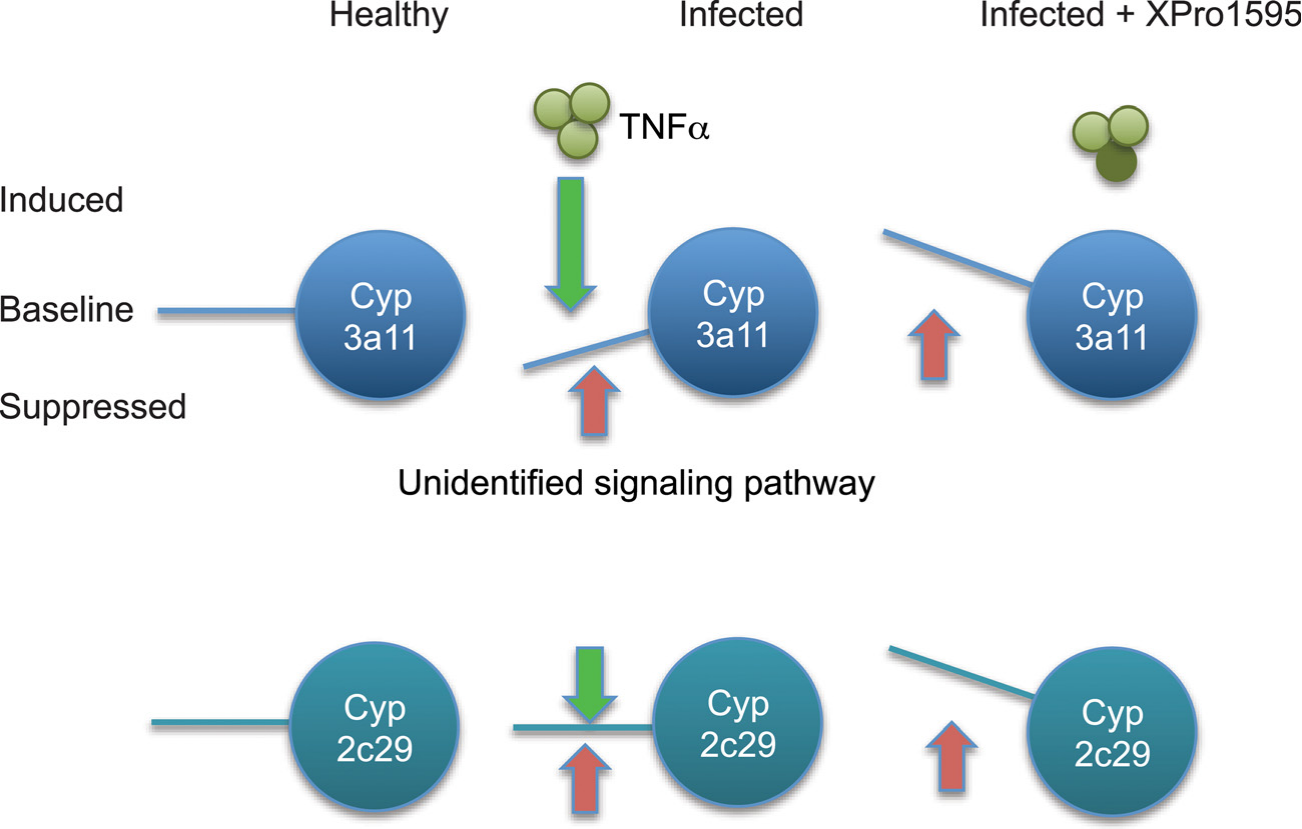
Proposed mechanism for disease-dependent upregulation of P450 enzymes by XPro1595 treatment.

Why were Cyp3a11 and Cyp3a25 mRNAs actually increased by infection in the animals treated with four doses of XPro1595? Similarly, Cyp2c29 and 3a13 mRNAs were only significantly increased in one or both of the XPro1595-treated groups (Fig. 4). It is well-documented that different P450s have different sensitivities to different inflammatory cytokines (reviewed in Morgan 1997). We can speculate that for Cyp3a11 and 3a25, there could be two opposing signals due to infection, of which the suppressive effect of TNF*α* is dominant (Fig. 7). For 2c29 and 3a13, the two signals cancel each other out. When the suppression by TNF*α* is removed, induction can occur. For other P450s, TNF*α* may not have an important in vivo role. It is possible that the increase in Cyp3a11 mRNA in mice that received four doses of XPro1595 is partly due to the apparent decrease in the corresponding uninfected group. However, this is not the case for Cyp3a25.

In the clear cases where XPro1595 had an effect on P450 regulation, the effect was greatest in the animals that received four doses, and less clear in the animals treated only once on day 7. This could be due to the need for an accumulation of XPro1595 with successive injections, or it could mean that TNF*α* begins to affect P450 expression before day 7, and those effects persist even when the effect of soluble TNF*α* is neutralized. As the single injection on day 7 had clear effects on hepatic IL6, TNF, and IL1*β* expression, there is again not a good correlation between overall effect on inflammation and on P450 regulation.

This study was designed to test the effect of XPro1595 on downregulation of P450s by infection, and therefore was not powered for multiple comparisons that would allow us to test the effect of XPro1595 alone on drug-metabolizing enzyme expression. Nevertheless, examination of the data (Figs. 3 and 4) suggests that the expression of Cyp2b10, Cyp4a10, and Cyp2c29 may be reduced by XPro1595 treatment; whereas Fmo3 expression may be increased. Additional studies will be needed to determine whether there are any significant effects on these enzymes in the absence of disease.

In conclusion, our results support previous work indicating a selective in vivo role for TNF*α* in the suppression of Cyp3a11 and Cyp3a25 in a model of acute infectious colitis. The ability of XPro1595 to abolish Cyp3a11 and Cyp3a25 suppression, and to uncover inductive effects of infection on Cyp3a11, 3a25, 2c29, and 3a13 in this model of inflammation suggests in principle that the propensities of biologic drugs to cause DDDI in humans may not be limited to just a normalization of decreased drug clearance due to inflammation. We speculate that DDDI could actually cause clearance of Cyp3a (CYP3A in humans) substrates such as statins, HIV protease inhibitors, cyclosporine etc., to increase above that found in the healthy state, with consequent decreases in efficacy of cotreated drugs in affected individuals. However, studies are needed in other disease models and with approved biologic drugs to determine whether or not this observation is generally applicable.

## Abbreviations

AGP: *α*_1_-acid glycoprotein
Cpr: NADPH-cytochrome P450 reductase
DDDI: disease-dependent drug–drug interaction
EDTA: ethylenediaminetetraacetic acid
EMIC: Emory Multiplexed Immunoassay Core
Fmo: flavin monooxygenase
GAPDH: glyceraldehyde-3-phosphate dehydrogenase
IFN*γ*: interferon-γ
IL: interleukin
LPS: lipopolysaccharide
mKC: chemokine (C-X-C motif) ligand 1
P450: cytochrome P450
RT-qPCR: reverse transcriptase-quantitative real-time polymerase chain reaction
SAA: serum amyloid A
SAP: serum amyloid P
TNFR1: TNF*α* receptor-1
TNF*α*: tumor necrosis factor-*α*

## Supporting Information

**Table S1.** Primer sequences. **Figure S1.** *Citrobacter rodentium* colony counts in liver, blood, and colon of infected mice Bacterial counts in colon and liver are expressed per g tissue, whereas counts in blood are per mL whole blood. Values represent mean colony forming units ±SEM. (*n* = 6). One-way ANOVA determined that there were no differences among the groups.
