# Supplementary material for: Selective effects of a therapeutic protein targeting tumor necrosis factor-alpha on cytochrome P450 regulation during infectious colitis: implications for disease-dependent drug–drug interactions
Source: Pharmacol Res Perspect. 2014 Feb 12;2(1):e00027. doi: 10.1002/prp2.27 (PMC3972819; doi:10.1002/prp2.27)

APPENDIX: SUPPORTING INFORMATION

Supplemental Table 1: Primer sequences

Gene name		Primers	Reference
GAPDH	Forward	AGCCAAACGGGTCATCATCTC	
	Reverse	GGGGGCTAAGCAGTTGGTG	
Fmo3	Forward	CAGCATTTACCAATCGGTCTTC	(Zhang et al., 2009)
	Reverse	TGACTTCCCATTTGCCAGTAG	
AGP	Forward	CTTCGGGAGTCTCAAACAATAGGT	(Richardson and Morgan, 2005)
	Reverse	GACAGTCCCCGTTTCTTCTCATCC	
SAP	Forward	AGGGCAGAGACAATGAGCTACT	(Shirai et al., 2009)
	Reverse	GGACTCCCAAGTGGTACATAGG	
SAA	Forward	GTAATTGGGGTCTTTGCC	(Lan et al., 2005) (Reigstad et al., 2009)
	Reverse	TTCTGCTCCCTGCTCCTG	
IL-1	Forward	CAACCAACAAGTGATATTCTCCATG	(Overbergh et al., 2003)
	Reverse	GATCCACACTCTCCAGCTGCA	
IL-6	Forward	CAACCACGGCCTTCCCTACT	(Richardson and Morgan, 2005)
	Reverse	TCATTTCCACGATTTCCCAGAG	
IFNγ	Forward	AACGCTACACACTGCATCTTGG	(Grabie et al., 2003) (Klein et al., 2005)
	Reverse	GCCGTGGCAGTAACAGCC	
TNFα	Forward	GACAAGGCTGCCCCGACTACG	(Richardson and Morgan, 2005)
	Reverse	CTTGGGGCAGGGGCTCTTGAC	
Cyp1a2	Forward	TGGAGCTGGCTTTGACACAG	(Pan et al., 2000)
	Reverse	CGTTAGGCCATGTCACAAGTAGC	
Cyp2a4/5	Forward	CTCGCCGAATTGTGGTGCTGTG	(Richardson and Morgan, 2005)
	Reverse	CCCGCTGCTGAAGGCTATGC	
Cyp2b10	Forward	CAGGTGATCGGCTCACACC	(Pan et al., 2000)
	Reverse	TGACTGCATCTGAGTATGGCATT	
Cyp2c29	Forward	AAGGTTTTGGCGTTGTTTTTAGC	(Richardson and Morgan, 2005)
	Reverse	AGCCTTTGGTTTTCCTCAGTTCTT	

Cyp2d9	Forward	CCTCCAAGGTCAGAAGTCCTTCA	(Blume et al., 2000)
	Reverse	CGATTTCTTGTTGGACTCTGCG	
Cyp2d22	Forward	GGGCCTTTGTTACCATGTTGG	(Blume et al., 2000)
	Reverse	TACTCGGCGCTGCACATCTG	
Cyp3a11	Forward	GGATGAGATCGATGAGGCTCTG	(Pan et al., 2000)
	Reverse	CAGGTATTCCATCTCCATCACAGT	
Cyp3a13	Forward	CCTCTGCCTTTCTTGGGGACGAT	(Richardson and Morgan, 2005)
	Reverse	CCGCCGGTTTGTGAAGGTAGAGT	
Cyp3a25	Forward	TTGGAGGCCTGAACTGCTAAAG	
	Reverse	CTGTGGGCCCTCATAAAACC	
Cyp4a10	Forward	TGTCCCAGGCATTGTCAGAGA	(Richardson and Morgan, 2005)
	Reverse	CCTTCGGGTTGTGGTGGAGA	
Cyp4a12	Forward	TGAGTCCTATGAAAGAGTGCC	(Nilakantan et al., 2008)
	Reverse	CTGGAAGCCCAGCAGAAGGTG	
Cyp4a14	Forward	CCCAAAGGTATCACAGCCACAA	(Richardson and Morgan, 2005)
	Reverse	CAGCAATTCAAAGCGGAGCAG	
Cyp4f18	Forward	AGAGCCTGGTGCGAACCTT	(Chaluvadi et al., 2009)
	Reverse	TGGAATATGCGGATGACTGG	

Blume N, Leonard J, Xu ZJ, Watanabe O, Remotti H, and Fishman J (2000) Characterization of Cyp2d22, a novel cytochrome P450 expressed in mouse mammary cells. Archives of Biochemistry and Biophysics 381 (2):191-204.

Chaluvadi MR, Kinloch RD, Nyagode BA, Richardson TA, Raynor MJ, Sherman M, Antonovic L, Strobel HW, Dillehay DL, and Morgan ET (2009) Regulation of hepatic cytochrome P450 expression in mice with intestinal or systemic infections of citrobacter rodentium. Drug Metabolism and Disposition 37 (2):366-374.

Grabie N, Hsieh DT, Buono C, Westrich JR, Allen JA, Pang H, Stavrakis G, and Lichtman AH (2003) Neutrophils sustain pathogenic CD8+ T cell responses in the heart. American Journal of Pathology 163 (6):2413-2420.

Klein RS, Lin E, Zhang B, Luster AD, Tollett J, Samuel MA, Engle M, and Diamond MS (2005) Neuronal CXCL10 directs CD8+ T-cell recruitment and control of West Nile virus encephalitis. Journal of Virology 79 (17):11457-11466.

Lan JG, Cruickshank SM, Singh JC, Farrar M, Lodge JP, Felsburg PJ, and Carding SR (2005) Different cytokine response of primary colonic epithelial cells to commensal bacteria. World Journal of Gastroenterology 11 (22):3375-3384.

Nilakantan V, Maenpaa C, Jia G, Roman RJ, and Park F (2008) 20-HETE-mediated cytotoxicity and apoptosis in ischemic kidney epithelial cells. American Journal of Physiology Renal Physiology 294 (3):F562-570.

Overbergh L, Giulietti A, Valckx D, Decallonne R, Bouillon R, and Mathieu C (2003) The use of real-time reverse transcriptase PCR for the quantification of cytokine gene expression. Journal of Biomolecular Techniques 14 (1):33-43.

Pan J, Xiang Q, and Ball S (2000) Use of a novel real-time quantitative reverse transcription-polymerase chain reaction method to study the effects of cytokines on cytochrome P450 mRNA expression in mouse liver. Drug Metabolism and Disposition 28 (6):709-713.

Reigstad CS, Lunden GO, Felin J, and Backhed F (2009) Regulation of serum amyloid A3 (SAA3) in mouse colonic epithelium and adipose tissue by the intestinal microbiota. PLoS One 4 (6):e5842.

Richardson TA and Morgan ET (2005) Hepatic cytochrome P450 gene regulation during endotoxin-induced inflammation in nuclear receptor knockout mice. Journal of Pharmacology and Experimental Therapeutics 314 (2):703-709.

Shirai R, Hirano F, Ohkura N, Ikeda K, and Inoue S (2009) Up-regulation of the expression of leucine-rich alpha(2)-glycoprotein in hepatocytes by the mediators of acute-phase response. Biochemical and Biophysical Research Communications 382 (4):776-779.

Zhang J, Chaluvadi MR, Reddy R, Motika MS, Richardson TA, Cashman JR, and Morgan ET (2009) Hepatic flavin-containing monooxygenase gene regulation in different mouse inflammation models. Drug Metabolism and Disposition 37 (3):462-468.

Supplemental Fig. 1. *Citrobacter rodentium* colony counts in liver, blood and colon of infected mice Bacterial counts in colon and liver are expressed per g tissue, whereas counts in blood are per ml whole blood. Values represent mean colony forming units ± S.E.M. (n=6). One-way ANOVA determined that there were no differences among the groups.

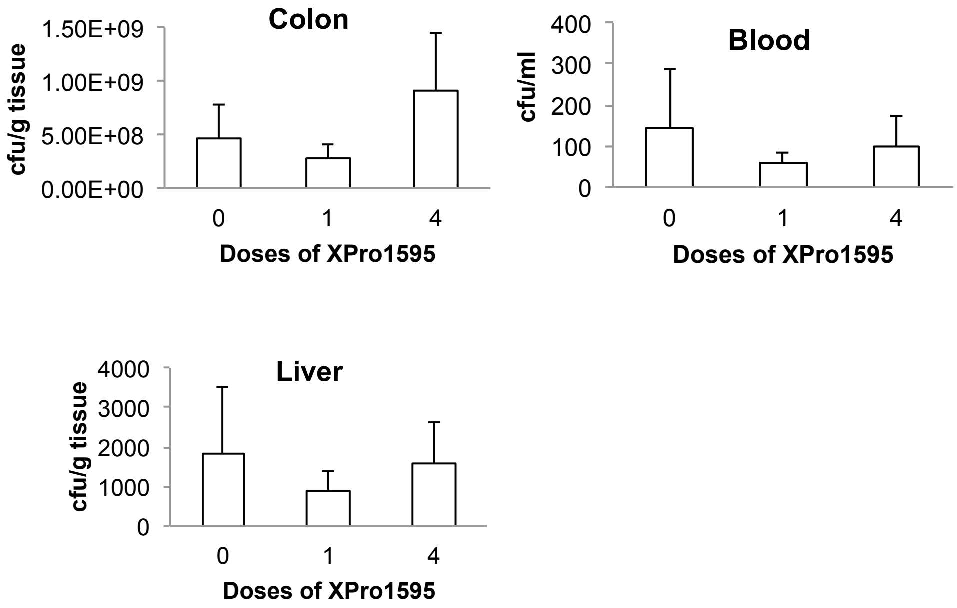

Supplement: Supplementary file 1 [file prp20002-e00027-SD1.pdf]
